# Efficient uremic toxins adsorption from simulated blood by immobilization of metal organic frameworks anchored Sephadex beads

**DOI:** 10.1038/s41598-025-92492-w

**Published:** 2025-03-20

**Authors:** Reda M. Abdelhameed, Mahmoud El-Shahat, Bahira Hegazi, Hassan Abdel-Gawad

**Affiliations:** 1 Applied Organic Chemistry Department, Chemical Industries Research Institute, National Research Centre, Scopus affiliation ID 60014618, 33 EL Buhouth St. , Dokki, Giza 12622 Egypt; 2 Photochemistry Department, Chemical Industries Research Institute, National Research Centre, Scopus affiliation ID 60014618, 33 EL Buhouth St., Dokki, Giza 12622 Egypt

**Keywords:** Sephadex, Metal-Organic framework, Toxin, Simulated blood, Adsorption, Kinetics, Isotherms, Environmental sciences, Chemistry

## Abstract

The current study outlines the removal of Creatinine, *p*-Cresol sulfate, and Hippuric acid from simulated blood using three new granules: Fe-BTC@Sephadex, Cu-BTC@Sephadex, and Co-BTC@Sephadex. Beads were used to adsorbed toxic chemicals, and the effects of various experimental parameters were examined in the adsorption optimization process. The framework’s adsorption isotherms were explained by the application of the Freundlich and Langmuir models. The kinetics of adsorption is represented by a pseudo-first and second-order equation. The morphology and structure of the Fe-BTC@ Sephadex, Co-BTC@ Sephadex, and Cu-BTC@Sephadex beads were investigated using Fourier transform infrared spectroscopy (FT-IR), scanning electron microscopy (SEM), and X-ray diffraction (XRD). The adsorption capacities for creatinine were 545.69, 339.76, and 189.88 mg/g for Fe-BTC@ Sephadex, Cu-BTC@ Sephadex, and Co-BTC@ Sephadex, respectively, according to the results; the corresponding adsorption capacities for hippuric acid were 323.78, 206.79, and 68.059 mg/g, and the maximum adsorption capacities for *p*-Cresol sulfate were 122.65, 71.268, and 40.347 mg/g, respectively. These were, in fact, promising findings that have implications for an industrial-scale transportable artificial kidney.

**Supplementary Information:**

The online version contains supplementary material available at 10.1038/s41598-025-92492-w.

## Introduction

Chronic kidney disease (CKD) is defined by a dynamic degradation of glomerular filtration, which results in the kidneys’ inability to remove potentially harmful chemicals from the blood circulation through the urine, causing a buildup of those molecules^1^. If these particles are naturally or chemically dynamic, they are referred to as uremic poisons; otherwise, they are known as uremic maintenance solutes^1^. A gradual endogenous inebriation and a dynamic worsening of the clinical circumstances are the results of the accumulation of these poisons, which have detrimental effects on the physiological capacity^1,2^. One potential primary uremic poison is creatinine, which when it builds up in the bloodstream can produce a number of detrimental symptoms that might reduce kidney function and hasten renal degradation^3^. The most prominent separating biomarkers of uremia are typically *p*-cresyl sulfate (PCS), indoxyl sulfate (IS), 3-carboxy-4-methyl-5-propyl-2-furanpropionic acid (CMPF), hippuric acid, and indole-3-acetic acid (IAA). These substances are thought to be prototype protein-bound uremic poisons, capable of binding over 90% of plasma proteins. Each of these four PBUTs possesses an ionic functional group and an aromatic ring. They can also create hydrophobic and electrostatic interactions, as well as non-covalent connections like hydrogen bonds and Van der Waals forces^4,5^. Adsorption-based separation techniques are now being investigated for the purpose of purifying blood^6^. Adsorbents in a column to enable a coordinated move between a dialysate or blood, and placing adsorbents at the layer surface were tested for uremic poison expulsion in order to develop wearable and adaptable devices^6^. Various adsorbent materials, including activated carbons, have been used to date.

^7,8^, zeolites^9–11^, carbon nanotubes^12^ and polymers^13–16^ have being investigated for fake kidney models. Because of its ultrahigh porosity and dynamic locations, metal-organic framework (MOF), a unique hybrid material with high thermal and chemical stability, has been shown to be more attractive than AC or mesoporous silica materials^17–19^. There have been previous theories regarding the inner MOF cage width and the BET surface area as crucial factors in MOFs’ ability to adsorb substances^20^. MOF is composed of organic linkers and metal ions that are joined by coordinate boundaries to form one-, two-, or three-dimensional structures^17,21^. Applications for MOFs have gained traction quickly, including but not limited to the separation of bioactive compounds^22^, water purification^23,24^, drug delivery^25^ and gas separation^26^. Nevertheless, MOF applications for artificial kidneys are still in their infancy. Furthermore, MOFs may be methodically investigated and integrated into a variety of functionalities, which sets them apart from other kinds of crystalline materials. They also have remarkable longevity^27^.

Metal–organic framework MIL-100(Fe) was investigated as a novel sorbent for counterfeit kidney application with a high adsorption capacity and remarkable reusability in order to eradicate creatinine as a common uremic toxin^28^. Using zirconium-based metal-organic frameworks (NU-1000), uremic poisons such as* P*-cresyl sulfate, indoxyl sulfate, and hippuric acid were removed from simulated blood. It demonstrated that human serum albumin had entirely eliminated *p*-cresyl sulfate^27^. The highest degree of adsorption of hippuric acid and 3-indoloacetic acid was achieved by UiO-66-NH_2_ (75%)^29^. Hippuric acid and indoxyl sulfate were eliminated using three Zr-MOFs: UiO-66, UiO-66-SO3H, and UiO-66-(COOH)_2_. For indoxyl sulfate, Zr-MOFs have an adsorption capacity of 49.5 mg g^− 1^, however for hippuric acid, that same capacity is 38.3 mg g^− 1^. Upon incorporating Zr-MOFs into the polylactide (PLA) matrix, 78% of indoxyl sulfate and 75% of hippuric acid were eliminated^30^. Because of its high adsorption capacity (190.5 mg/g), simple desorption, and excellent reusability, MIL100(Fe) may be an extremely promising sorbent for the removal of creatinine^28^. To create UiO-66-(COOH)_2_@ cotton fabric composite, UiO-66-(COOH)2 was directly implanted as MOF materials within cotton fabric. Maximum creatinine adsorption capacities of the resultant composites were found to be 113.6 mg/g for pristine tissue and 212.8 mg/g for UiO-66-(COOH)_2_@tissue composite, respectively^31^.

A cross-linked dextrin gel called Sephadex is utilized in gel filtration. After Jerker Borath and Per Flodden worked on its development, Pharmacia released it in 1959 ^32^. The name is derived from **se**paration Pharmacia dextran. It is most frequently used for gel filtration columns and is typically produced as beads. Modification of the degree of cross-linking can alter the gel’s fractionation properties. The minuscule beads used in these very gel filtration and chromatographic media are produced artificially from the polysaccharide dextrin. A three-dimensional network with ionic functional groups connected to the glucose units in the saccharide chains via ether bonds is created by cross-linking the organic chains. Anion and cation exchangers and gel filtration resins with varying porosities are included in accessible forms; bead sizes are in distinct ranges from 20 to 300 μm. Another application for sephadex is in ion-exchange chromatography. The isolation and identification of flavonoids from certain plant sources has been made easier by new technology, but traditional techniques—particularly Sephadex^®^ LH-20 (SLH)-have been widely employed because they are affordable, practical, quick, and efficient^33^. A study was conducted on the purine adsorption process in Sephadex G-10 chromatography^34^. A new technique based on SephadexTM for eliminating lingering cytotoxic and microbicidal agents when evaluating disinfectants against viruses: Studies investigated utilizing the human coronavirus as a model.

^35^.Despite the fact that MOF nanoparticles are very advantageous, their capacity to regenerate and recover is limited by their incapacity to readily separate from the aqueous phase during operation. Consequently, MOF nanoparticles need to be combined with substrate materials in real-world applications. Studies using MOFs as adsorbents in movable artificial kidneys are, nevertheless, comparatively uncommon. Considering the aforementioned, it would be advantageous to immobilize MOF nanoparticles on substrate materials for use as adsorbents in portable artificial kidneys^36^.The objective of this study was to decide how well beads, which are inexpensive and environmentally acceptable adsorbents, work in removing Creatinine, *p*-Cresol sulfate and Hippuric acid from simulated blood. The kinetics and isotherm parameters were also assessed utilizing the adsorption data.

## Materials and methods

### Materials

Sephadex^®^ G-100 was obtained from sigma. Acetic acid glacial (CH_3_COOH, ≥ 99.5% purity) was of reagents grade from the Greagent. Ethyl alcohol (CH_3_COCH_3_ ≥ 99.7%) was purchased from General-Reagent; 1, 3, 5-tricarboxylic benzene (98%), Iron (III) nitrate nonahydrate (Fe (NO_3_)_3_·9H_2_O ≥ 99.0%), Copper (II) nitrate trihydrate (Cu (NO_3_)_2_·3H_2_O ≥ 99.0%), Cobalt (II) nitrate hexahydrate (Co (NO_3_)_2_·6H_2_O ≥ 99.0%), and methyl alcohol (CH_3_OH, ≥ 99.9%) were all acquired from sigma. Toxins creatinine, *p*-cresol sulfate and hippuric acid were all purchased from sigma.

### Preparation of adsorbents

**Fig. 1:**
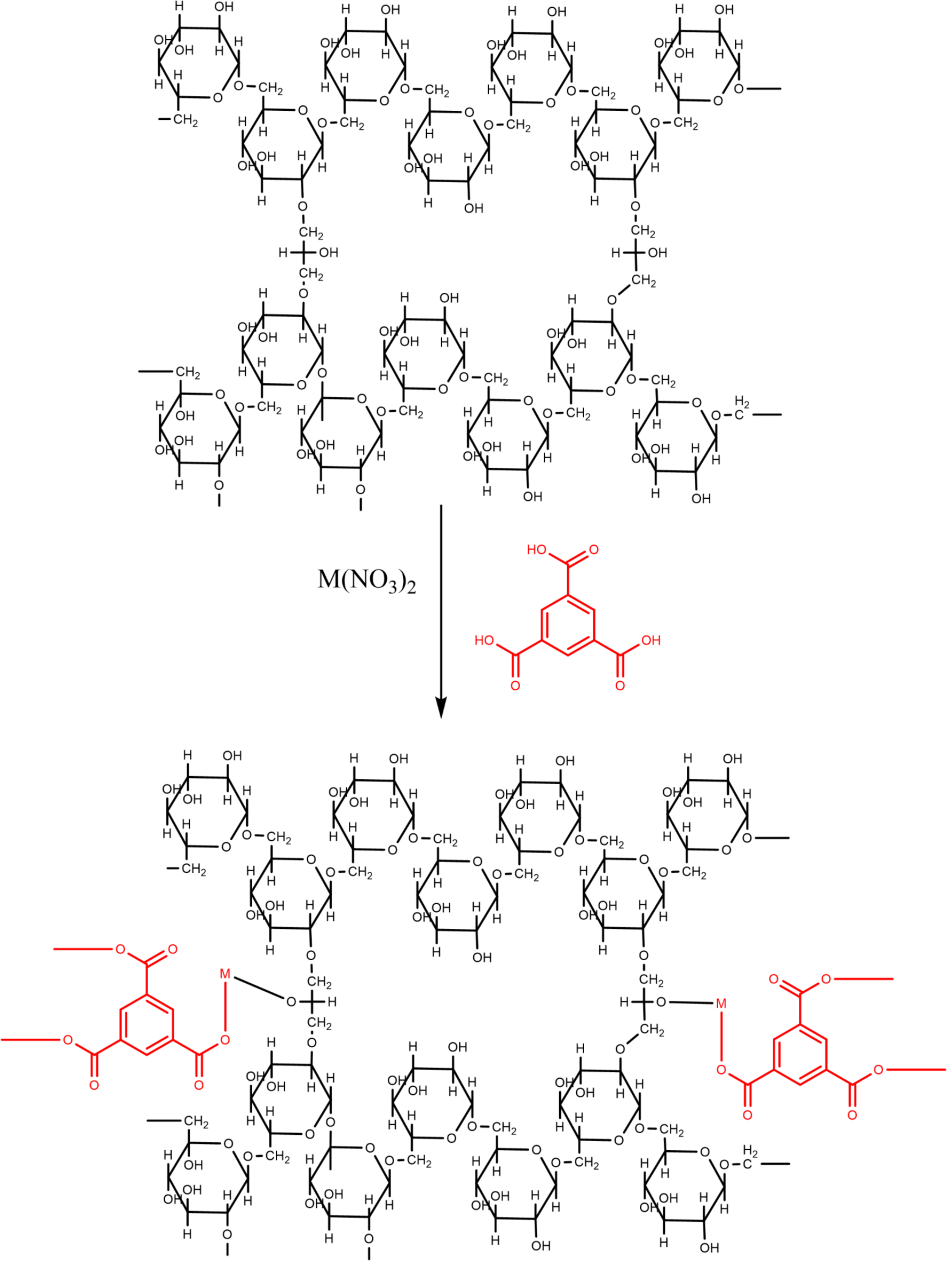
Preparation of Fe-BTC@ Sephadex, Cu-BTC@ Sephadex and Co-BTC@ Sephadex adsorbents.

Figure 1 depicts the general procedures for synthesis porous MOFs onto sephadex. Fe-BTC@ Sephadex was synthesized as follow: Initially, 1.0 g of sephadex was added by magnetic stirring at room temperature to 50 mL of DMF. Then, iron (III) nitrate nonahydrate (0.404 g, 1 mmol) was added to the dispersed sephadex, heated in an oven for two hours at 50 °C, and the resulting solids were filtered out and then cleaned with methanol. Subsequently, 1, 3, 5-tricarboxylic benzene (0.21 g, 1 mmol) was dissolved in DMF and added to Fe@sephadex. The mixture was then dispersed using ultrasonic dispersion for 60 min. The mixture was then heated at 100 °C for at least 15 h. The solids were separated and washed with methanol several times and dried on open air oven at 50 °C for 2 h.

Cu-BTC@Sephadex was prepared as follow: sephadex (1.0 g) and copper (II) nitrate trihydrate (0.241 g, 1 mmol) were dispersed on 50 mL DMF then heated at 50 °C for 2 h, the solids were separated and washed several time with methanol and dried. Finally, 1, 3, 5-tricarboxylic benzene (0.21 g, 1 mmol) was dissolved in DMF and added to Cu@sephadex. The mixture was added on oven at 100 °C for 15 h, then the mixture was cold down and the solids were separated and washed with methanol and dried on the oven at50°C for 2 h.

Co-BTC@ Sephadex was prepared using the same process, sephadex (1.0 g) and cobalt (II) nitrate hexahydrate (0.291 g, 1 mmol) were dispersed on 50 mL DMF then heated at 50 °C for 2 h, the solids were separated and washed with methanol and dried. Finally, 1, 3, 5-tricarboxylic benzene (0.21 g, 1 mmol) was dissolved in DMF and added to Co@sephadex. Methanol was used to purify the finished product.

### Characterization of materials

The morphological structures of the Fe-BTC@, Co-BTC@, and Cu-BTC@ beads were examined using high resolution scanning electron microscopy (HRSEM Quanta FEG 250 with field emission gun, FEI Company - Netherlands). The elemental analysis was recorded using an energy dispersive X-ray (EDX) spectroscopy instrument (EDAX Analyzer AME-TEK) connected to the same microscope. X-ray diffraction (XRD) could be observed for THE samples under Cu K X-radiation at 40 kV, 50 mA, and = 1.5406) at room temperature. Diffraction data were collected in steps of 2° ranging from 4° to 50° with a step size of 0.02° and scanning rate of 1 s. Fourier Transform Infrared Spectroscopy (FTIR) examination was performed on the materials using a Japanese JASCO FT/IR-4700 spectrophotometer during the inspection process.

## Results and discussion

### Characterization of nanocomposite

#### XRD and FTIR studies

**Fig. 2:**
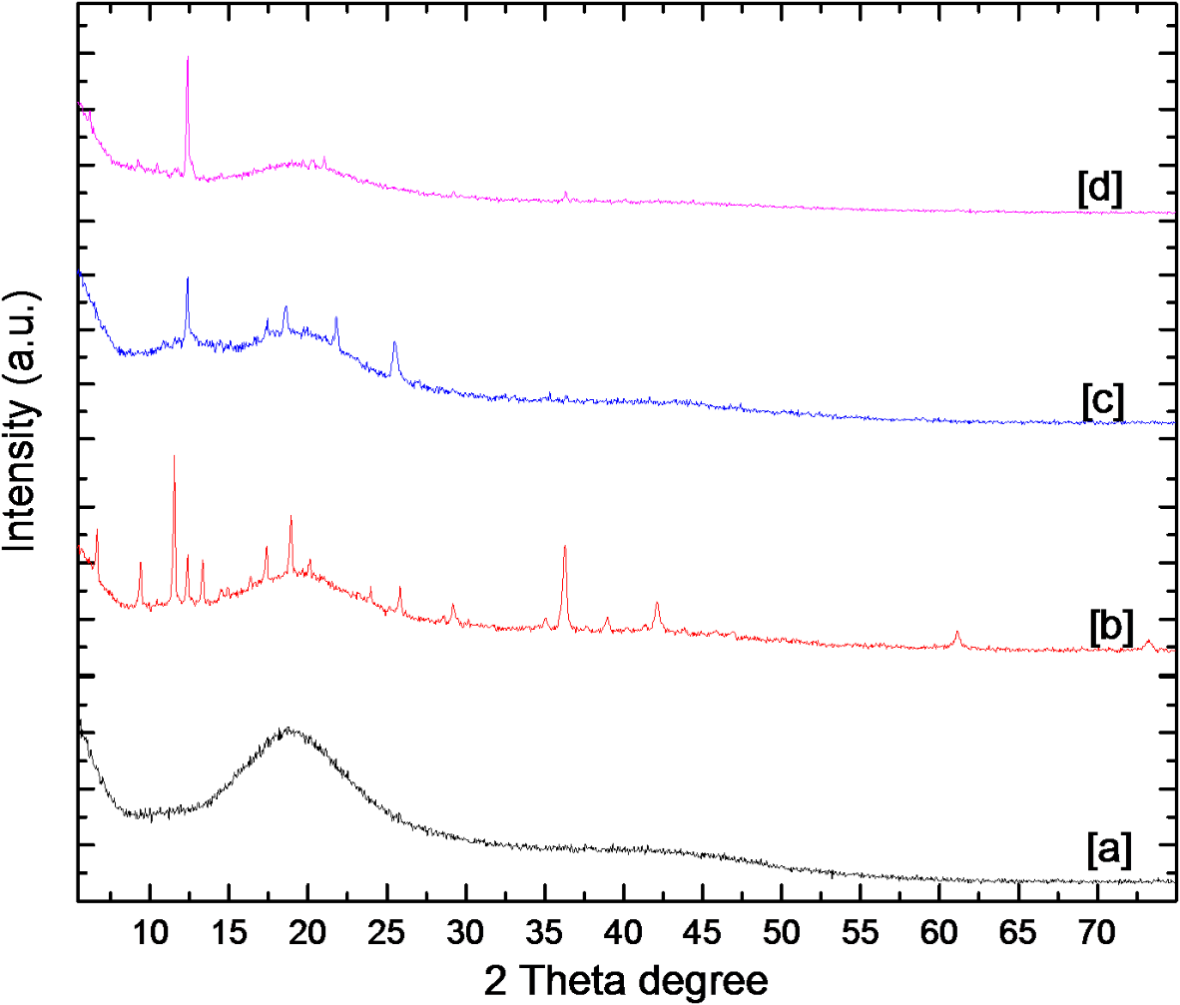
PXRD of (**a**) Sephadex (**b**) Fe-BTC@ Sephadex, (**c**) Co-BTC@ Sephadex and (**d**) Cu-BTC@Sephadex.

The XRD spectra of the nanomaterials are displayed in Fig. 2, where a distinctive broad peak at 19.69° is displayed by sephadex. 7.3°, 10.4°, 12.7°, 14.7°, 16.5°, 18.0°, 22.1°, 24.5°, and 26.7° were the diffraction peaks of the prepared Fe-BTC@ Sephadex, Cu-BTC@ Sephadex, and Co-BTC@ Sephadex. These peaks correspond to the crystal surfaces (0 1 1), (0 0 2), (1 1 2), (0 2 2), (0 1 3), (2 2 2), (1 1 4), (2 3 3) and (1 3 4). The acquired data show the presence of M-BTC, however there is only one diffraction peak in the case of Cu-BTC@Sephadex due to its extremely low content.

**Fig. 3:**
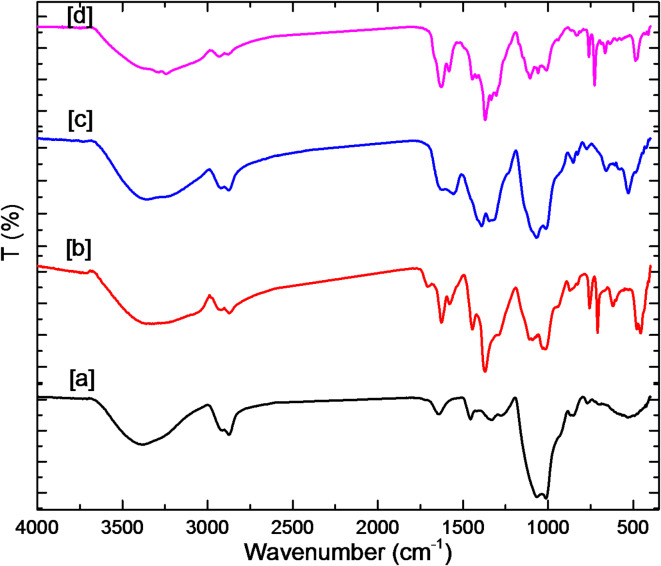
FTIR of (**a**) Sephadex (**b**) Fe-BTC@ Sephadex, (**c**) Co-BTC@ Sephadex and (**d**) Cu-BTC@Sephadex.

Fe-BTC@ Sephadex, Cu-BTC@ Sephadex, and Co-BTC@ Sephadex were all characterized using FT-IR spectroscopy (Fig. 3, Figures S1-12). A large number of hydroxyl groups are present in the sephadex structure, whereas M-BTC is created when metal M coordinates with the O atom in the BTC molecule. A large and noticeable band was discovered at 3433 cm^− 1^ in the FTIR spectra of sephadex, which was found to originate from the O–H stretching band. The C–H stretching is represented by the absorption peak at 2876 cm^− 1^, whereas the C–O–C stretching is represented by the peaks at 1653, 1598, and 1080 cm^− 1^. The distinctive peaks at 2924 cm^− 1^ for M-BTC result from the aromatic ring on BTC being stretched, whereas the stretching vibration of C = O is responsible for the bands at 1577 and 1137 cm^− 1^. The M-O stretching is indicated by the peak at 423 cm^− 1^. FTIR spectroscopy provided some insights into the structural characteristics of M-BTC, sephadex, and the composite M-BTC@sephadex. The bands seen at 1350 ∼ 1500, 900 ∼ 1350, and 500 ∼ 800 cm^− 1^ are caused by the benzene rings’ stretching, plane, and out-of-plane vibrations. When combined, these distinctive peaks show that M-BTC forms on the surface of sephadex.

#### SEM and EDS analysis

**Fig. 4:**
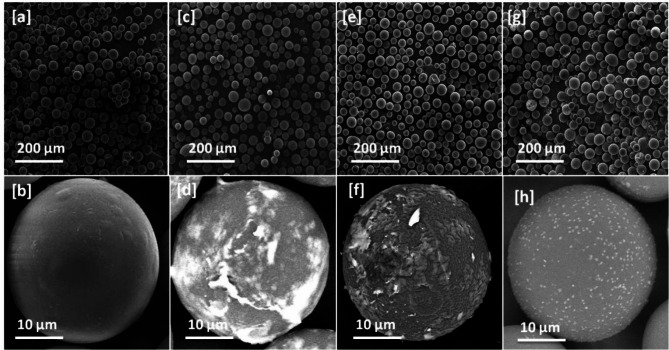
SEM of (**a**, **b**) Sephadex (**c**, **d**) Fe-BTC@ Sephadex, (**e**, **f**) Co-BTC@ Sephadex and [g, h] Cu-BTC@Sephadex.

**Fig. 5:**
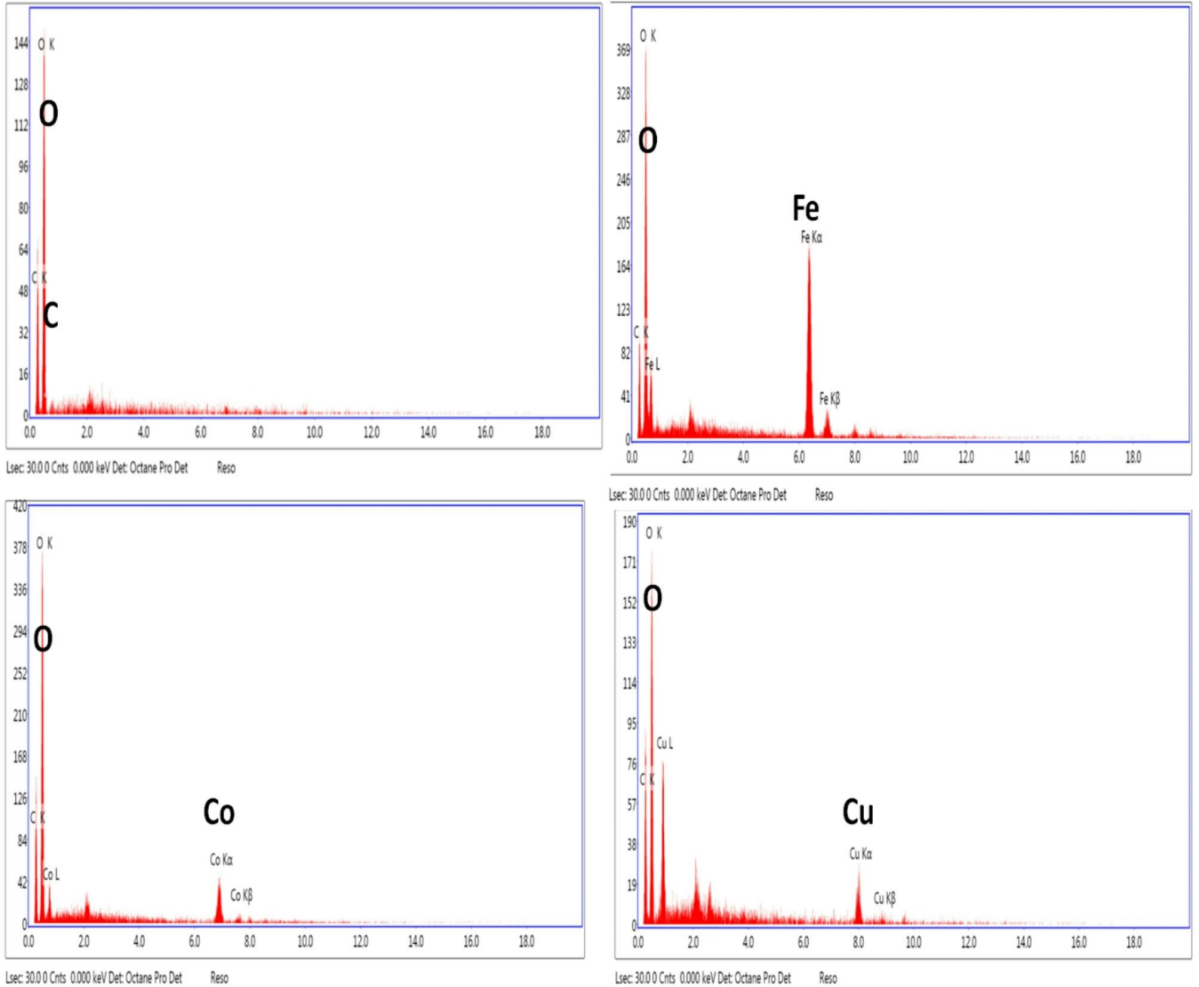
EDX of (**a**) Sephadex (**b**) Fe-BTC@ Sephadex, (**c**) Co-BTC@Sephadex and (**d**) Cu-BTC@Sephadex.

Fe-BTC@Sephadex, Cu-BTC@Sephadex, and Co-BTC@Sephadex FESEM microscopic pictures are displayed in Fig. 4, demonstrating the effective coating of the outer layer of the sephadex structure with M-BTC. Sephadex has a comparatively smooth structure with a layered makeup. The major component of Fe-BTC@Sephadex, Cu-BTC@Sephadex, and Co-BTC@Sephadex is a single crystal structure with a homogeneous size distribution. The chemical makeup of the produced Fe-BTC@ Sephadex, Cu-BTC@ Sephadex, and Co-BTC@ Sephadex composites was displayed in the EDS spectra (Fig. 5).

The fundamental components of sephadex are represented by the conspicuous diffraction peaks in Fig. 5, which confirm the presence of oxygen (O) and carbon (C). The integration of sephadex into the Fe-BTC in the Fe-BTC@Sephadex complex, which was successfully absorbed into the material, is confirmed by an extra Fe diffraction peak. These samples showed no additional impurity peak in any images because of their excellent purity and single-phase development. Furthermore, the Cu-BTC@Sephadex and Co-BTC@Sephadex composites were produced efficiently. The EDX image of M-BTC included the appearance of the C, O, Co, and Cu elements.

### Adsorption isotherms

**Fig. 6:**
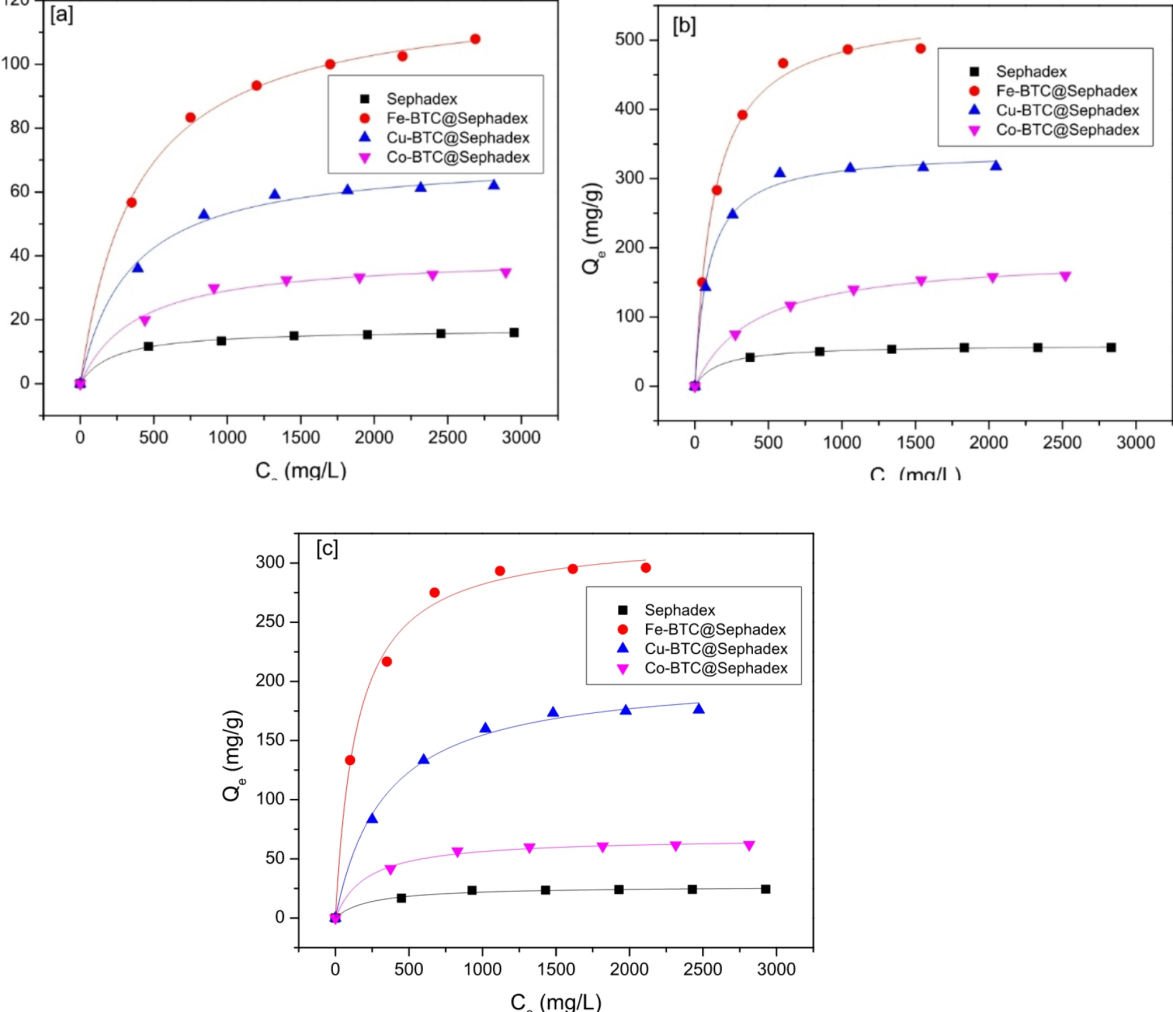
Langmuir isotherm of (**a**) *p*-Cresol sulfate on to Sephadex, Fe-BTC@Sephadex, Cu-BTC@Sephdex and Co-BTC@Sephadex; (**b**) Creatinine on to sephadex, Fe-BTC@Sephdex, Cu-BTC@Sephdex and Co-BTC@Sephadex; (**c**) Hippuric acid on to Sephadex, Fe-BTC@Sephadex, Cu-BTC@Sephadex and Co-BTC@Sephadex.

To investigate the impact of adsorption on Creatinine, *p*-Cresol sulfate, and Hippuric acid from simulated blood, adsorption tests were conducted for various adsorbents. Figure 6 illustrates this effect’s particular outcomes. It is evident that Fe-BTC@ Sephadex, Cu-BTC@ Sephadex, and Co-BTC@ Sephadex had better adsorption efficacy than sephadex alone when tested under the same experimental conditions. Additionally, creatinine shows better adsorption on Fe-BTC@ Sephadex, Cu-BTC@ Sephadex, and Co-BTC@ Sephadex. This could be because creatinine is a smaller molecule than hippuric acid and *p*-Cresol sulfate, making it easier to occupy the active site. As a result, Fe-BTC@Sephadex, Cu-BTC@Sephadex, and Co-BTC@Sephadex exhibit notable creatinine selectivity.

**Fig. 7:**
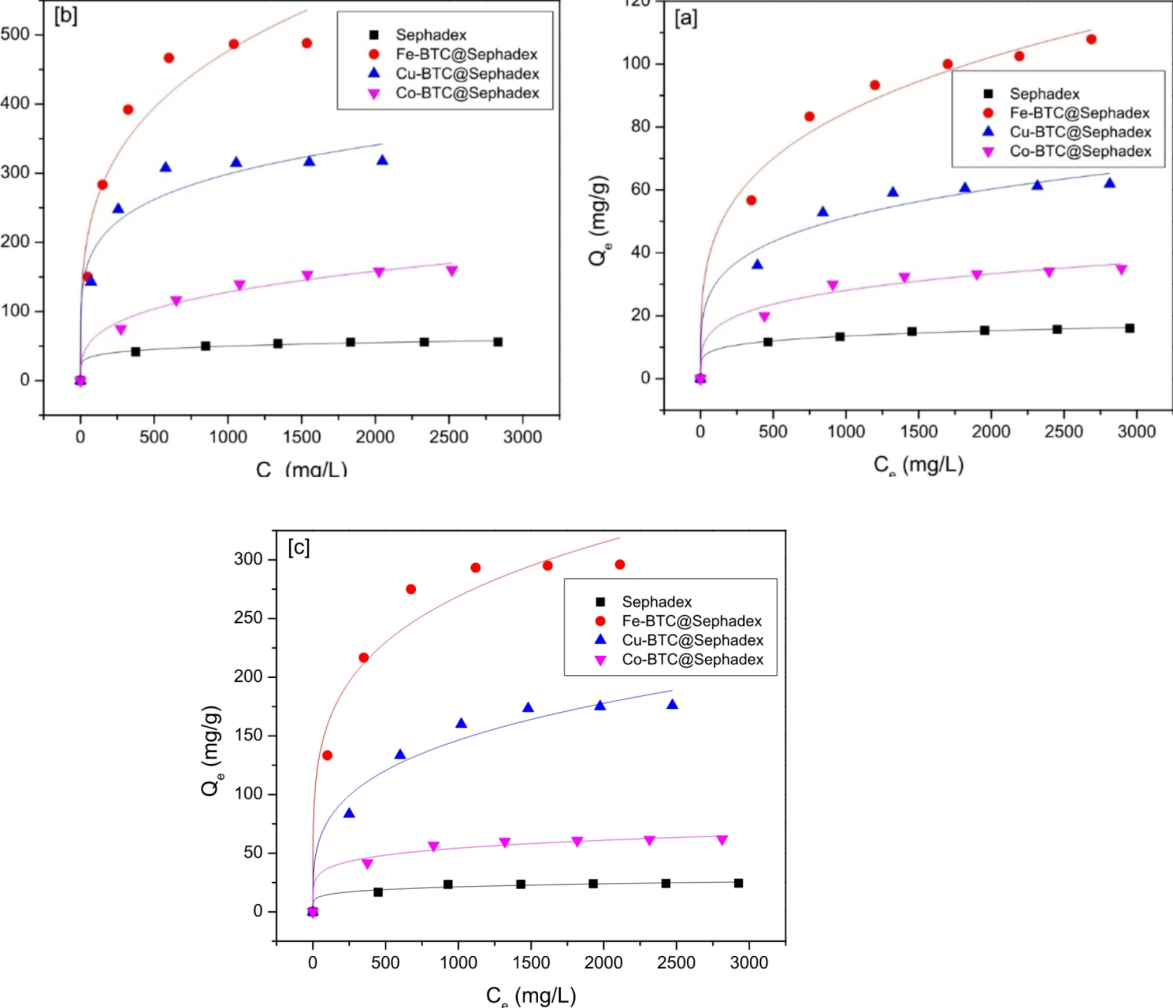
Freundlich isotherm of (**a**) *p*-Cresol sulfate on to Sephadex, Fe-BTC@Sephadex, Cu-BTC@Sephdex and Co-BTC@Sephadex; (**b**) Creatinine on to Sephadex, Fe-BTC@Sephdex, Cu-BTC@Sephdex and Co-BTC@Sephadex; (**c**) Hippuric acid on to Sephadex, Fe-BTC@Sephadex, Cu-BTC@Sephadex and Co-BTC@Sephadex.

Table 1; Fig. 6 show the fitting curves and calculation parameters of the two models on the test data. Compared to sephadex; Fe-BTC@, Cu-BTC@, and Co-BTC@ exhibit superior adsorption efficacy for creatinine, *p*-Cresol sulfate, and hippuric acid. Figure 7 shows that the adsorption process of creatinine, *p*-Cresol sulfate, and hippuric acid by Fe-BTC@ Sephadex, Cu-BTC@ Sephadex, and Co-BTC@ Sephadex composites was clearly more compatible with the Langmuir model than the Freundlich model because R^2^ in the case of Langmuir model was ranged between 0.999 and 0.987 and χ^2^ value was low but in the case of Freundlich model R^2^ ranged between 0.949 and 0.951 and χ^2^ value was high. The adsorption capacity of *p*-Cresol sulfate exhibited 17, 40, 71, and 122 mg/g, onto sephadex, Co-BTC@ Sephadex, Cu-BTC@Sephadex, and Fe-BTC@ Sephadex, respectively. The maximum creatinine adsorption capacity onto sephadex, Co-BTC@ Sephadex, Cu-BTC@Sephadex, and Fe-BTC@ Sephadex reached to 59, 189, 339, and 545 mg/g, respectively. The adsorption uptake capacities of hippuric acid onto sephadex, Co-BTC@ sephadex, Cu-BTC@ sephadex, and Fe-BTC@ sephadex were 26, 68, 206, and 323 mg/g, respectively. The prepared composites were ordered in terms of adsorption capacities as follow: Fe-BTC@Sephadex > Cu-BTC@Sephadex > Co-BTC@Sephadex and the toxin molecules selectivity as follow: creatinine > Hippuric acid > *p*-Cresol sulfate.

**Table 1:** Parameters of isotherm for the three toxins adsorption onto Fe-BTC@ Sephadex, Cu@Sephadex@BTC, and Co@Sephadex@BTC beads.

Sample	Langmuir				Freundlich			
	K_L_ (mL mg)	Q_m_ (mg g^− 1^)	*R* ^2^	χ^2^	*n*	K_F_	*R* ^2^	χ^2^
Creatinine								
Sephadex	0.0062	59.704	0.999	0.175	7.11	18.887	0.954	2.42
Fe-BTC@ Sephadex	0.0077	545.69	0.996	130.1	3.66	72.269	0.941	2081.1
Cu-BTC@ Sephadex	0.0108	339.76	0.995	64.60	5.27	80.607	0.945	809.3
Co-BTC@Sephadex	0.0025	189.88	0.998	5.753	3.27	15.521	0.958	75.2
*p*-Cresol sulfate								
Sephadex	0.00432	17.152	0.997	0.067	5.86	4.1661	0.956	0.09
Fe-BTC@ Sephadex	0.0026	122.65	0.998	2.41	3.65	12.774	0.944	22.1
Cu -BTC@ Sephadex	0.00295	71.268	0.992	3.64	4.27	10.164	0.951	14.6
Co-BTC@Sephadex	0.00258	40.347	0.991	1.29	4.06	5.1139	0.942	4.39
Hippuric acid								
Sephadex	0.0043	26.971	0.987	0.993	6.08	6.8395	0.951	2.29
Fe-BTC@ Sephadex	0.0068	323.78	0.995	63.1	4.39	55.839	0.962	477.5
Cu-BTC@ Sephadex	0.0029	206.79	0.995	21.3	3.56	21.032	0.966	144.6
Co-BTC@Sephadex	0.0047	68.059	0.994	2.67	5.91	16.867	0.949	10.62

### Adsorption kinetics

**Fig. 8:**
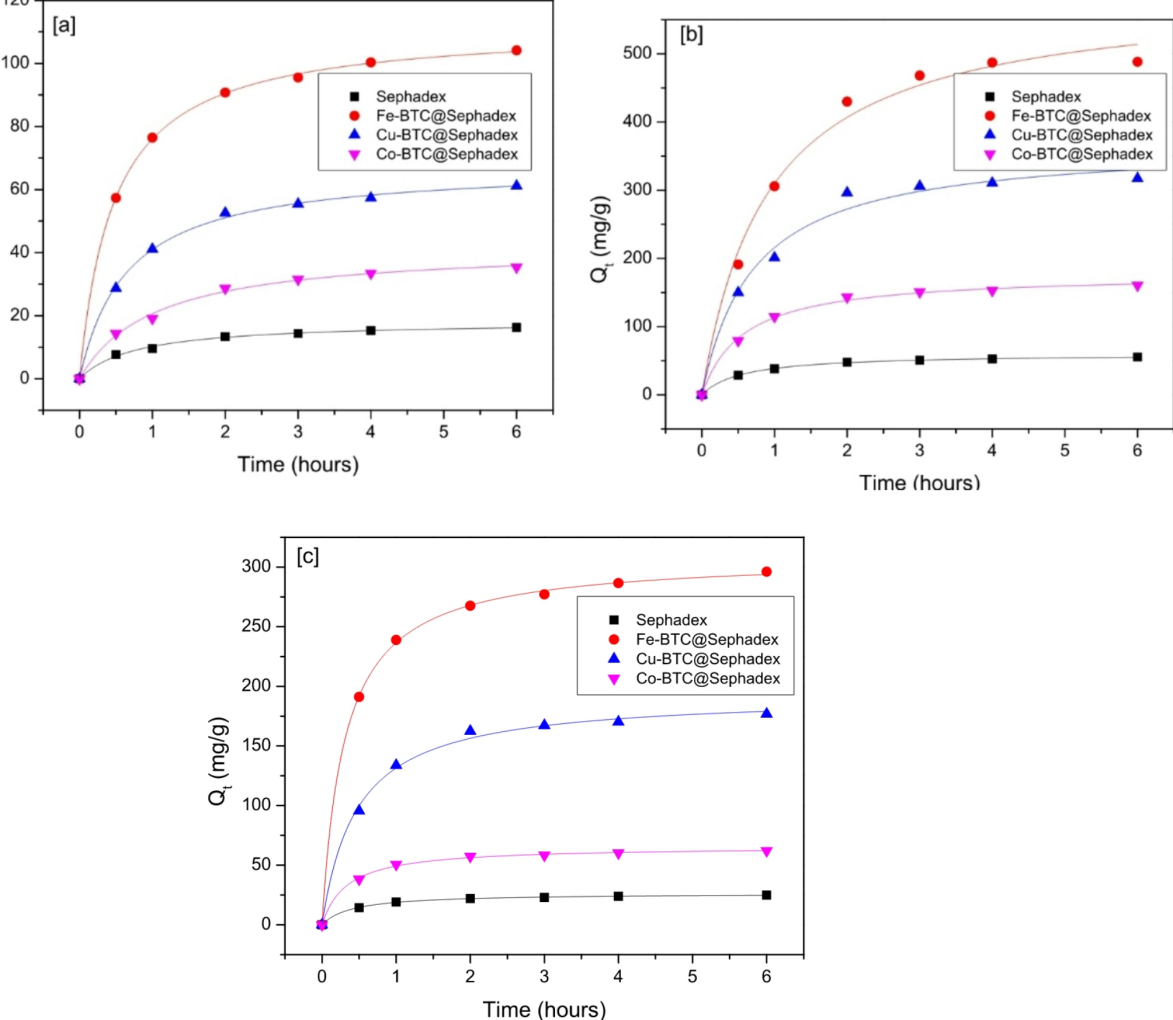
Kinetic studies of (**a**) *p*-Cresol sulfate on to Sephadex, Fe-BTC@Sephadex, Cu-BTC@Sephdex and Co-BTC@Sephadex; (**b**) Creatinine on to sphadex, Fe-BTC@Sephdex, Cu-BTC@Sephadex and Co-BTC@Sephdex; (**c**) Hippuric acid on to Sephadex, Fe-BTC@Sephadex, Cu-BTC@Sephadex and Co-BTC@Sephadex. Pseudo-second order molding fitting.

The effect of contact time on the adsorption capacity of creatinine, *p*-Cresol sulfate, and hippuric acid was investigated in order to evaluate the adsorbent’s efficacy in adsorbing these three substances. Figure 8 displays the results of kinetic testing and model fitting for hippuric acid, *p*-Cresol sulfate, and creatinine with different adsorbents. The results show that Co-BTC@, Cu-BTC@, and Fe-BTC@ exhibit superior adsorption performance for creatinine,* p*-Cresol sulfate, and hippuric acid compared to sephadex. According to the findings, the adsorption effectiveness rose quickly within the first few moments of contact time.

**Fig. 9:**
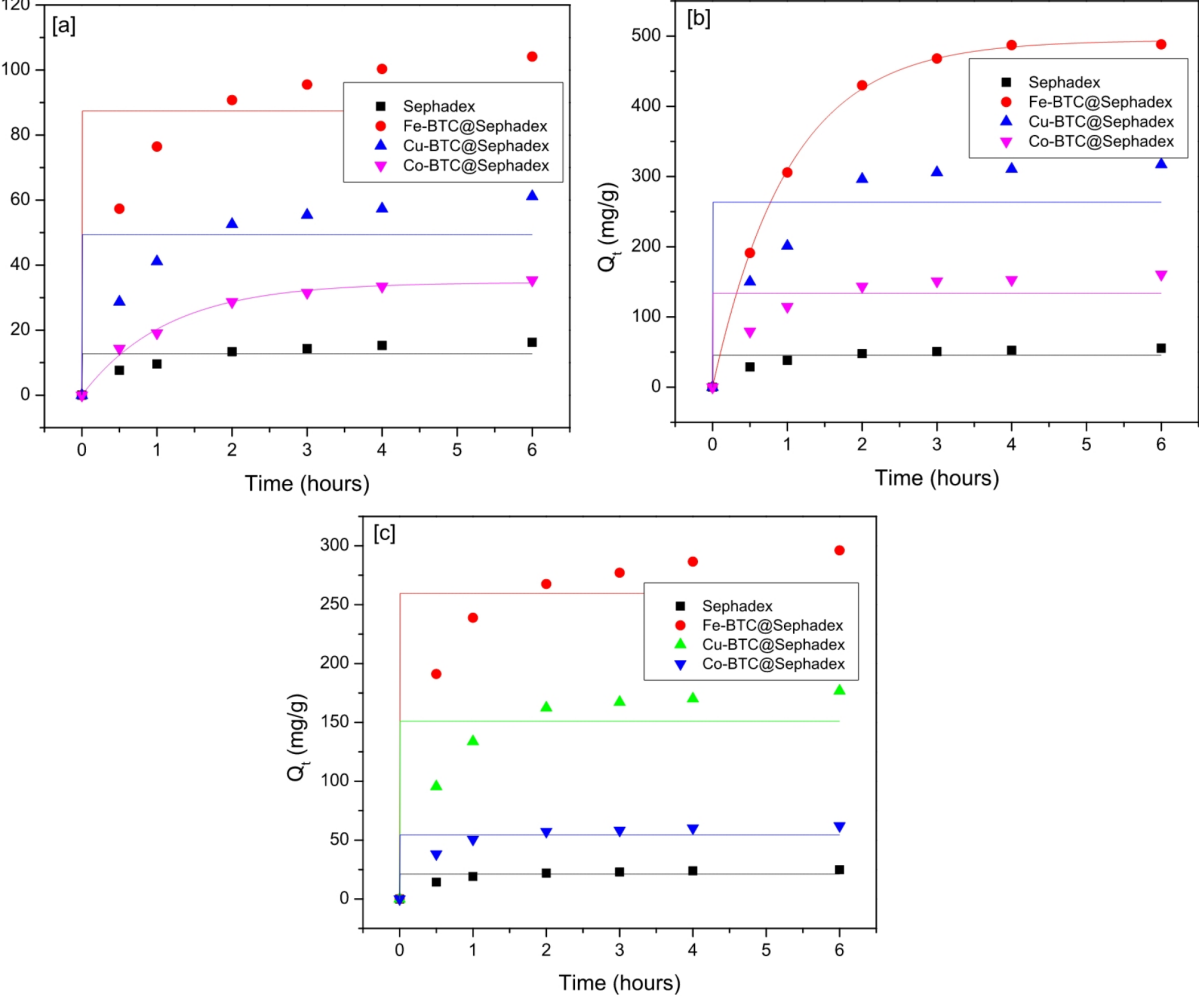
Kinetic studies of (**a**) *p*-Cresol sulphate on to Sephadex, Fe-BTC@Sephadex, Cu-BTC@Sephdex and Co-BTC@Sephadex; (**b**) Creatinine on to sphadex, Fe-BTC@Sephdex, Cu-BTC@Sephadex and Co-BTC@Sephdex; (**c**) Hippuric acid on to Sephadex, Fe-BTC@Sephadex, Cu-BTC@Sephadex and Co-BTC@Sephadex. Pseudo-first order molding fitting.

The degree of absorption then increased more slowly before stabilizing, suggesting that hippuric acid, *p*-Cresol sulfate, and creatinine diffuse into the solution quickly and stick to the surface of the adsorbent. As the duration of contact increases, the adsorbent’s active sites eventually disappear. The mass transfer barrier between the solid and the liquid increases as the concentration of the solution drops, weakening the adsorption force and lowering the adsorption efficiency value. Both pseudo-first-order (PFO) and pseudo-second-order (PSO) kinetic models were used to analyze the adsorption mechanism. Figures 8 and 9 displayed the nonlinear representations of the PFO and PSO models. Based on the non-linear equations’ intercept and slope, the values of k_1_, k_2_, and Q_e_ were determined. The PSO model exhibits a good degree of fit to the experimental data (R^2^ > 0.998). The parameters of the fitting curves of the PFO and PSO models were analyzed in relation to the dynamic parameters (Table 2) and the experimental data (Figs. 8 and 9). Thus, it can be said that the chemisorption process was the main proposed mechanism for this adsorption process.

**Table 2:** Kinetic parameters for the three toxins adsorption onto Fe-BTC@ Sephadex, Cu@Sephadex@BTC, and Co@Sephadex@BTC beads.

Sample	Pseudo-first-order				Pseudo-second-order			
	K_1_ (min^− 1^)	Q_e (cal)_ (mg g^− 1^)	*R* ^2^	χ^2^	K_2_ (g mg^− 1^ min^− 1^)	Q_e (cal)_ (mg g^− 1^)	*R* ^2^	χ^2^
Creatinine								
Sephadex	15556.0	45.541	0.730	103.21	0.0296	60.32	0.999	0.1785
Fe-BTC@ Sephadex	0.9829	494.13	0.999	13.302	0.0018	591.5	0.989	358.06
Cu-BTC@Sephadex	47923.0	263.43	0.647	4946.6	0.0038	369.1	0.985	203.77
Co-BTC@ Sephadex	47347.0	133.59	0.713	959.70	0.0098	177.9	0.996	10.837
*p*-Cresol sulfate								
Sephadex	984.23	12.738	0.647	11.562	0.0699	18.25	0.994	0.1689
Fe-BTC@ Sephadex	39937.0	87.420	0.770	309.90	0.0189	111.9	0.999	0.3611
Cu-BTC@Sephadex	12087.0	49.363	0.684	149.21	0.0225	67.74	0.998	0.7175
Co-BTC@ Sephadex	0.8788	34.684	0.991	1.3155	0.0229	42.03	0.995	0.8108
Hippuric acid								
Sephadex	11867.0	21.178	0.803	15.122	0.0933	26.37	0.999	0.0576
Fe-BTC@ Sephadex	18568.0	259.55	0.861	1512.2	0.0106	308.7	0.999	4.3087
Cu-BTC@Sephadex	75315.0	150.95	0.763	958.46	0.0111	192.9	0.996	14.266
Co-BTC@ Sephadex	37891.0	54.458	0.839	78.502	0.0456	65.68	0.997	1.0535

### Comparison of adsorption of uremic toxin with other adsorbents

The comparison of the various adsorbents’ highest uremic toxin elimination capacities can be found in Table 3. Fe-BTC@Sephadex has a significant deal of potential for numerous uremic detoxifications, as is evident. For the adsorption of creatinine, the corresponding adsorption capacities for hippuric acid were 323.78, 206.79, and 68.059 mg/g, while the maximum adsorption capacities for *p*-Cresol sulfate were 122.65, 71.268, and 40.347 mg/g, respectively. The adsorption capacities for Fe-BTC@ Sephadex, Cu-BTC@Sephadex, and Co-BTC@ Sephadex were 545.69, 339.76, and 189.88 mg/g, respectively. For similar session preparation and maximum absorption capabilities, a number of published papers pertaining to the sorption of creatinine, hippuric acid, and *p*-Cresol sulfate onto different adsorbent materials have been compiled. Using chitosan/sericin biopolymer membranes, even lower concentrations of creatinine (100.5–212 mg/g) were eliminated^31,37^. The utilization of Zeolites ZSM-5 and Hollow fiber membrane resulted in the lowest amount of adsorbed creatinine (6.22–86.2/g), respectively^38,39^. The ease of use of the present work is attributed to its one-pot synthesis method, little chemical consumption, lack of organic solvent usage, and easily applied compound resembling a filter.

**Table 3:** Comparison of adsorption capacity of *p*-cresol sulfate, creatinine and Hippuric acid on Fe-BTC@ Sephadex, Cu-BTC@Sephadex, and Co-BTC@Sephadex adsorbents.

Adsorbents	Maximum adsorption capacity (mg/g)			References
	*p*-Cresol sulfate	Creatinine	Hippuric acid	
Fe-BTC@ Sephadex	122.65	545.69	323.78	This work
Cu-BTC@Sephadex	71.268	339.76	206.79	This work
Co-BTC@Sephadex	40.347	189.88	68.059	This work
amine-functionalized mesoporous silica	–	542.6	–	^40^
Hollow fiber membrane	–	86.2	–	^39^
Zeolites ZSM-5	–	6.22	–	^38^
Zr-MOFs	–	–	38.3	^41^
UAPNFM	–	168.63	–	^42^
Zr-based MOF	–	212.8	–	^31^
ZJU-X6	197.2	–	–	^43^
ZJU-X7	57	–	–	^43^
Silicalite	106	–	–	^44^
NU-1000	440	–	–	^27^
PPNUH	282	–	–	^45^
Fe_3_O_4_/MOF/IBU	25.5	–	–	^46^
MIL-100 (Fe)	15.5	–	–	^20^
biopolymers of chitosan/sericin (CS/SS) composite nanofibers	–	100.5	–	^37^

### Adsorption mechanism

The adsorption mechanism relies on different strategies, one of these strategies is to form a complex between toxin molecules and a metal ion center on the prepared composite, the three working composite have different metal center, iron III, copper II and cobalt II in the backbone of the network, it well known that iron III prefers coordination number 6, however, copper(II) and cobalt(II) prefer coordination number 4, therefore, iron located on Fe-BTC@Sephdex can coordinate with more toxin molecules and enhance the adsorption capacity rather than two other adsorbent materials. However, the geometry and surface area of ​​the prepared composite play an important role in the adsorption of toxins, here, Fe-BTC@Sephdex has a higher surface area than Cu-BTC@Sephadex and Co-BTC@Sephadex, therefore, the adsorption capacity was enhanced in the case of Fe-BTC@Sephdex. Creatinine uptake onto Sephadex, Fe-BTC@Sephadex, Cu-BTC@Sephadex, and Co-BTC@Sephadex adsorbents were higher than *p*-Cresol sulfate and hippuric acid because increases in electron-donating capacity at ring nitrogens for creatinine rather than other toxins leading to facilitate the coordination with the metal center in the composites.

**Fig. 10:**
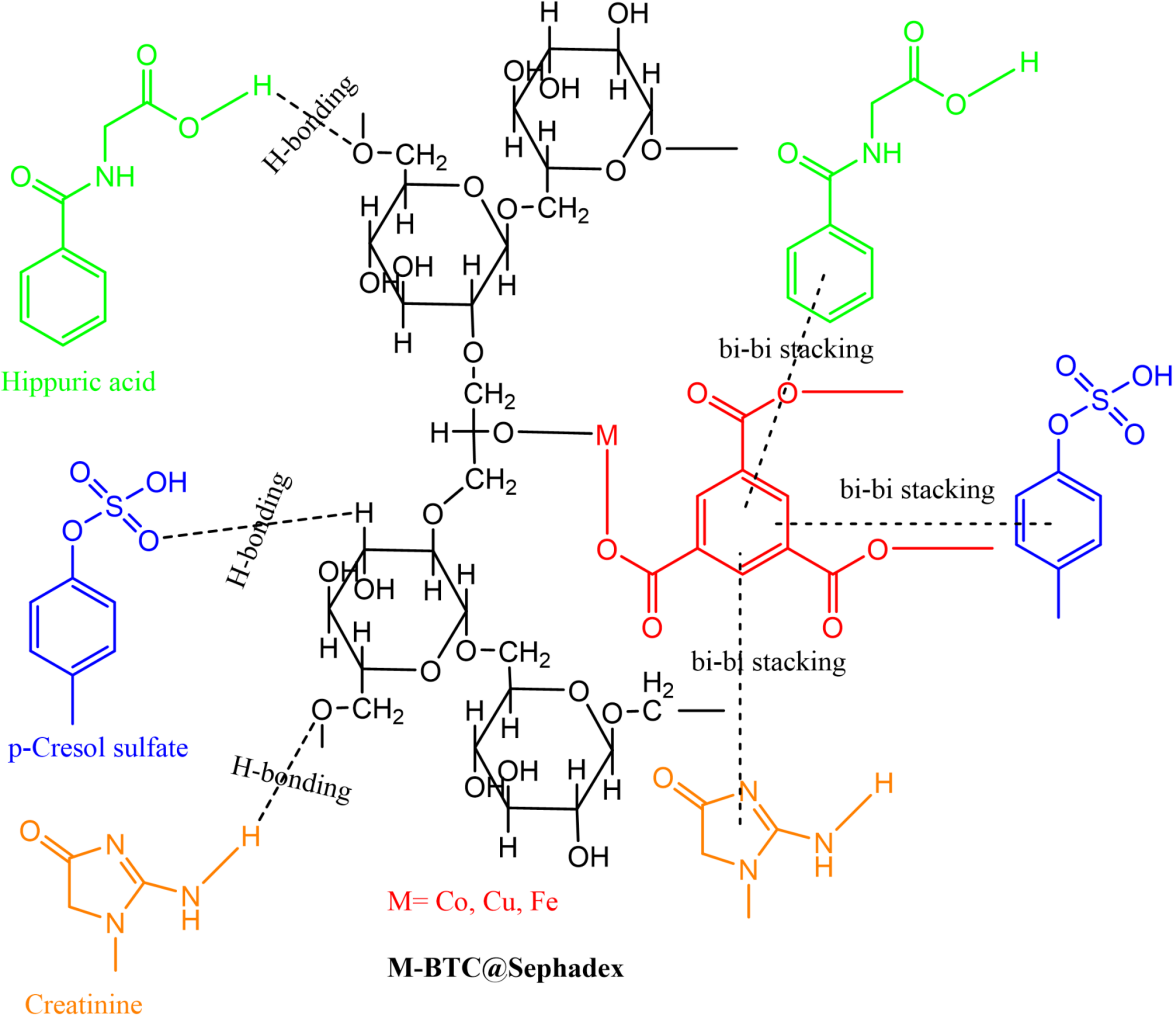
Proposed mechanism of toxins adsorption using M-BTC@Sephadex.

Hydrogen bonds were the second option for the adsorption mechanism proposal, creatinine, *p*-Cresol sulfate, and hippuric acid have nitrogen with unshared electron pairs and Sephadex, Fe-BTC@ Sephadex, Cu-BTC@Sephadex, and Co-BTC@Sephadex adsorbents have hydroxyl groups with free electrons on oxygen atoms, these items can correlate via hydrogen bonding. Furthermore, the free electron on the free hydroxyl groups in the composite can form hydrogen bonds with the hydrogen of creatinine or amino groups. Additionally, dipole-dipole, dipole-induced dipole, and dispersion forces might potentially be involved in the adsorption of toxin molecules on composites because of the polarizability of z-electrons and dipole moments of toxin molecules. The possible mechanism for the removal of toxins mainly depends on π-π interaction because the two systems used (adsorbent and adsorbate) have π system. Pore filling was among the several interactions that facilitated the adsorption of creatinine, *p*-Cresol sulfate, and hippuric acid onto metal-organic framework-based sephadex (MOFs@ Sephadex)^47,48^. All possible mechanisms were proposed in Fig. 10.

## Conclusions

Using the solvothermal approach, Fe-BTC@Sephadex, Cu-BTC@Sephadex, and Co-BTC@Sephadex were successfully synthesized. The resulting Fe-BTC@Sephadex, Cu-BTC@Sephadex, and Co-BTC@Sephadex composites were used as adsorbents for creatinine, *p*-Cresol sulfate, and hippuric acid from simulated blood after being subjected to many instrumental evaluations. Research indicates that the adsorption behavior of sephadex may be considerably impacted by the inclusion of MOFs. The effects of important variables on the adsorption process, such as contact time and the starting concentrations of hippuric acid, *p*-Cresol sulfate, and creatinine from simulated blood, were investigated.

According to the results of kinetics and isotherms, creatinine, *p*-Cresol sulfate, and hippuric acid can be adsorbed on Fe-BTC@Sephadex, Cu-BTC@Sephadex, and Co-BTC@Sephadex using the Langmuir isotherm model and PSO kinetic model. The developed compound’s remarkable elimination efficiency points to its possible application as a dialysis therapy agent.

## Electronic supplementary material

Below is the link to the electronic supplementary material.

Supplementary Material 1

## Data Availability

The all data generated or analyzed during the current study are included in this manuscript.
