# Supplementary material for: Efficient uremic toxins adsorption from simulated blood by immobilization of metal organic frameworks anchored Sephadex beads
Source: Sci Rep. 2025 Mar 20;15:9667. doi: 10.1038/s41598-025-92492-w (PMC11926176; doi:10.1038/s41598-025-92492-w)
Supplement: Supplementary file 1 — Supplementary Material 1 [file 41598_2025_92492_MOESM1_ESM.docx]

**Supporting information**

**Batch Adsorption experiments**

Adsorption studies were conducted in 15 mL glass flasks with different experimental concentration settings and reaction times. The following effects of toxin concentrations were investigated: During the combination studies, varied concentrations (100–1500 mg L^–1^) of creatinine, *p*-Cresol sulfate, and hippuric acid were mixed with 2.5 g L^–1^ of Fe-BTC@ Sephadex, Co-BTC@ Sephadex, and Cu-BTC@Sephadex beads at 303 K for up to 6 hours.

Throughout the experiment, sample flasks were removed from the reaction shaking table and centrifuged at regular intervals. There were three duplicate experiments carried out. The absorbance of creatinine, *p*-Cresol sulfate, and hippuric acid was measured independently at wavelengths of 268 nm, 277 nm, and 270 nm using a UV spectrophotometer (JASCO). The concentration of the combined pollutants before and after the reaction was determined using the peak area and calibration curve.

The amounts of the three poisons eliminated by Fe-BTC@ Sephadex, Co-BTC@ Sephadex, and Cu-BTC@ Sephadex beads under varied circumstances were calculated using Equation 1:

| $Q_{t}= \frac{{(C}_{o}-C_{t})V}{m}$ | (1) |
| --- | --- |

Where t is the duration of the three toxins' adsorption and Q_t_ (mg g^-1^) is the amount of Fe-BTC@, Co-BTC@, and Cu-BTC@ beads adsorption concentration, The symbols C0, Ct (mg L^-1^), and t stand for the initial, residual, and temporal concentrations of toxins, respectively. The liquid volumes (L) and masses (g) of the Fe-BTC@, Co-BTC@, and Cu-BTC@ beads are denoted by the letters V and m, respectively. The elimination effectiveness (R) of each of the three poisons is determined by Eq. (2).

| $\% R= \frac{{(C}_{o}-C_{t})}{C_{o}} 100$ | (2) |
| --- | --- |

This is how the impact of time on toxin absorption was examined: The effect of varying time intervals (0.5 to 6 hours) was investigated at fixed concentrations of the three toxins (500 mg L^-1^) and fixed concentrations of beads (2.5 g L^-1^). The temperature was also held constant at 303 K.

The adsorption kinetics of the three toxins were investigated using kinetic investigation theory, which was founded on pseudo-first- and pseudo-second-order kinetic models (Table 1). The pseudo-first order kinetic model is expressed as follows in Equation 3:

$\ln\left( q_{e}-q_{t} \right)=lnq_{e}-k_{1}t$ (3)

Where the rate constant of the pseudo-first-order kinetic process is denoted by k_1_ (min^-1^). The quantity of toxins that have been adsorbed at time t (min), time q_e_ (mg g^-1^), and time q_t_ (mg g^-1^), in that order.

Equation 4 represents the pseudo-second-order kinetic model.

| $q_{t}=\frac{k_{2}q_{e}^{2}t}{{1+k}_{2}\mathrm{tq}_{e}}$ | (4) |
| --- | --- |

In the case of different Fe-BTC@ Sephadex, Co-BTC@ Sephadex, and Cu-BTC@ Sephadex beads adsorbents, the adsorption capacities at equilibrium at time (t) are denoted by q_e_ and q_t_, respectively, and the rates constants of the pseudo-first-order and pseudo-second-order kinetic models are denoted by k_1_ and K_2_.

The Langmuir and Freundlich models served as the foundation for the adsorption isotherm theory, and it has been demonstrated that the Langmuir model is a nonlinear equation (Eq. 5):

| $Q_{e}= \frac{Q_{m}k_{L}C_{e}}{1+ k_{L}C_{e}}$ | (5) |
| --- | --- |

Where Q_e_ is the equilibrium adsorption capacity, Q_m_ is the greatest metal-organic framework modified by sephadex adsorption capacity, C_e_ is the equilibrium concentration of the three poisons, and K_L_ is the Langmuir constant. Conversely, the Freundlich experimental model (Eq. 6) does not make any assumptions about surface adsorption and instead treats adsorption as heterogeneous.

| $Q_{e}= k_{F}C_{e}^{\frac{1}{n}}$ | (6) |
| --- | --- |

Where n is an empirical value, K_F_ is the Freundlich adsorption equilibrium constant, C_e_ is the equilibrium concentration of the three poisons, and Q_e_ is the equilibrium adsorption capacity. The 1/n has a value between 0 and 1 and indicates how concentration affects adsorption strength.

**FT-IR spectroscopy**

**Figure S1:** [a] Sephadex, [b] Cresol sulfate and [c] Cresol sulfate@Sephadex

**Figure S2:** [a] Fe-BTC@Sephadex, [b] Cresol sulfate and [c] Cresol sulfate@Fe-BTC@Sephadex

**Figure S3:** [a] Co-BTC@Sephadex, [b] Cresol sulfate and [c] Cresol sulfate@Co-BTC@Sephadex

**Figure S4:** [a] Cu-BTC@Sephadex, [b] Cresol sulfate and [c] Cresol sulfate@Cu-BTC@Sephadex

**Figure S5:** [a] Sephadex, [b] Hippuric acid and [c] Hippuric@Sephadex

**Figure S6:** [a] Fe-BTC@Sephadex, [b] Hippuric acid and [c] Hippuric@Fe-BTC@Sephadex

**Figure S7:** [a] Co-BTC@Sephadex, [b] Hippuric acid and [c] Hipuric@Co-BTC@Sephadex

**Figure S8:** [a] Cu-BTC@Sephadex, [b] Hippuric acid and [c] Hippuric@Cu-BTC@Sephadex

**Figure S9:** [a] Sephadex, [b] Creatinine and [c] Creatinine@Sephadex

**Figure S10:** [a] Fe-BTC@ Sephadex, [b] Creatinine and [c] Cratinine@Fe-BTC@Sephadex

**Figure S11:** [a] Co-BTC@ Sephadex, [b] Creatinine and [c] Creatinine@Co-BTC@Sephadex

**Figure S12:** [a] Cu-BTC@ Sephadex, [b] Creatinine and [c] Creatinine@Cu-BTC@Sephadex
